# Challenges and Ongoing Actions to Address the Mpox Emergency in Africa

**DOI:** 10.5334/aogh.4580

**Published:** 2024-11-15

**Authors:** Faraan O. Rahim, Mosoka Fallah, Urvish Jain, Eugene T. Richardson, Nicaise Ndembi, Ngashi Ngongo, Jean Kaseya

**Affiliations:** 1 Harvard Medical School, Harvard University, Boston, MA, USA; 2 Africa Centres for Disease Control Prevention (Africa CDC), Addis Ababa, Ethiopia; 3 School of Medicine, University of Pittsburgh, Pittsburgh, PA, USA; 4 Brigham and Women’s Hospital, Boston, MA, USA

**Keywords:** mpox, Africa, global health

## Abstract

This review examines key events, challenges, and responses to the mpox public health emergency following the Africa CDC’s declaration of a Public Health Emergency of Continental Concern on August 13, 2024. In response to the crisis, over 3.6 million vaccine doses and more than $150 million in funding have been mobilized globally, with contributions from the United States, European Union, and Japan. However, challenges persist, particularly in the Democratic Republic of Congo, where a humanitarian crisis in Kinshasa has complicated mpox diagnostics and treatment. In response, the Africa CDC has deployed its One Continental Incident Management Support team, with a focus on decentralizing diagnostics and enhancing sample movement through additional PCR equipment, funded by the Pandemic Fund and USAID. To reinforce laboratory diagnostics, surveillance, and case management, the Africa CDC has adopted a comprehensive “One Team, One Plan, One Budget, One M&E” approach and has deployed 72 epidemiologists to improve data integration. Collaborative efforts with WHO, GAVI, and UNICEF aim to expedite vaccine distribution, with a target of 10 million doses by 2025, alongside enhanced vaccine safety monitoring.

Between 1 January and 3 November 2024, a total of 46,794 suspected mpox cases and 844 deaths were reported across 19 African countries [1]. The 2024 mpox outbreak, marked by its rapid spread and severity, began to escalate in early April, when cases initially centered in the Democratic Republic of Congo (DRC) started spreading to other countries, including Burundi, Kenya, and Rwanda—nations previously unaffected by mpox [2]. With case numbers tripling compared with the 2022 outbreak and a high case fatality rate, the Director General of the Africa Centres for Disease Control (CDC) declared the mpox outbreak a Public Health Emergency of Continental Security (PHECS) on 13 August 2024. Just 24 hours later, the World Health Organization (WHO) Director declared mpox a Public Health Emergency of International Concern (PHEIC) [3].

Over the several weeks since, Africa CDC has helped coordinate regional, national, and continental efforts to curb the spread of the mpox virus in Africa. The United States, European Union via Health Emergency Preparedness and Response Authority (HERA), GAVI, the United Nations Children’s Fund (UNICEF), Japan, and Canada have pledged to donate over 6.1 million mpox vaccines, with a large portion to the mpox epicenter in DRC [4]. Additionally, initial donations of $10.4 million from the African Union (AU) and $10 million from the US Agency for International Development (USAID) have now been supplemented by $500 million from the United States and $128.89 million Fast‑Track Allocation from the Pandemic Fund to combat mpox in 10 African Countries [5, 6]. Africa CDC, WHO, and other multilateral organizations have also formed the Mpox Continental Preparedness and Response Plan to mobilize these resources and strengthen mpox preparedness in DRC, Burundi, Cameroon, the Central African Republic, Côte d’Ivoire, Gabon, Liberia, Kenya, Nigeria, Rwanda, South Africa, and Uganda [7].

Despite swift global efforts, the international response to mpox faces significant challenges. A major obstacle is the political instability and ongoing humanitarian crisis displacing over 1.6 million people in the North and South Kivu region of DRC [8–10]. Underdeveloped health infrastructure, combined with concurrent outbreaks of cholera, widespread food insecurity, sexual violence, and mass displacement have made it difficult to detect and respond to the mpox outbreak in the epicenter of the global emergency [11].

To address the crisis, the Africa CDC and WHO have implemented the “One Budget, One Plan, and One M&E Framework” and established the “One Continental Incident Management Support Team” based in Kinshasa, DRC [7]. This 50‑member team is coordinating efforts through weekly meetings with National Public Health Institute directors and partners, providing updates on the mpox epidemic situation and response strategies. The team leverages funding from AU, international partners, and domestic resources from the DRC and Burundi governments to bolster local responses, including enhancing surveillance and laboratory testing capabilities. As part of this initiative, 20,000 mpox testing cartridges, quantitative polymerase chain reaction (qPCR), and other essential laboratory supplies are being procured for affected countries. The Africa CDC is also working with UNICEF, which has launched a $58.8 million appeal to provide humanitarian support to children and communities disproportionately affected by mpox, particularly in DRC and Burundi [12].

Another significant challenge in the global response to mpox is the limited availability of effective therapeutics. A recent study indicated that while antiviral medications like tecovirimat are safe, they did not significantly improve the resolution of clade I mpox in DRC [13]. Currently, critical drugs for managing symptoms like skin rashes and pain relief are the primary standard of care, which complicates definitive treatment efforts across African countries. To address these gaps, the Africa CDC Mpox Research Coordination team held a continental research and development conference on 29 August 2024 in Kinshasa. The conference aimed to close knowledge gaps in medical countermeasures and foster collaboration between the Africa CDC and global research and pharmaceutical organizations to develop effective mpox therapeutics.

Furthermore, limited mpox diagnostic equipment and surveillance tools pose significant challenges. There are no point‑of‑care diagnostics for clade I mpox, making it difficult to diagnose, isolate in a timely manner, and provide supportive treatment closer to infected individuals [14]. The gold standard PCR test requires reagents and a reliable electricity supply, both of which are lacking in remote areas. Moreover, the clinical sensitivity against PCR in DRC has been low and there have been reports of serious issues with cross reactivity with other orthopox and non‑orthopox viruses [15]. Inadequate community surveillance and the spread of infodemics further hinder response efforts. Additionally, the siloed nature and lack of robust data infrastructure integration across provinces within DRC and in other affected countries such as Burundi, CAR, Congo, and Rwanda have complicated continental surveillance of infections, the tracking of hospital admissions and deaths, and the targeting of vaccinations.

In response, the Africa CDC, in collaboration with WHO, is working to decentralize diagnostics by leveraging existing infrastructure from WHO and the US President’s Emergency Plan for AIDS Relief and facilitating the transport of samples in regions where point‑of‑care diagnostics are unavailable [16]. In the coming months, they also plan to leverage the Pandemic Fund and USAID funds to procure more PCR machines and lab diagnostic equipment to improve mpox diagnosis. There is a plan to strengthen community alert systems and surveillance to enhance active surveillance and contact tracing. Each affected country has submitted a cost response plan, which has been integrated into the unified One Team, One Plan, One Budget, One M&E framework [7].

Furthermore, several African countries have activated certain levels of laboratory diagnostics, surveillance, case management, coordination, and Risk Communication and Community Engagement (RCCE). Additionally, surge capacity has been bolstered with Africa CDC’s deployment of 72 epidemiologists to affected AU states [7]. These efforts are beginning to yield results, helping outline the epidemiological situation in each country. The goal is to ensure that local health systems can collect and share data within their designated jurisdictions, facilitating data integration across countries to enhance continental surveillance and help guide public health officials in implementing more targeted policies.

The distribution of mpox vaccines is a critical component of the response to mpox in African nations. Several countries have committed to donating vaccines, with the United States and European nations—including Spain, Germany, and France—pledging 600,000 doses of the MVA‑BN vaccine, while Japan has committed to donating 3 million doses of the LC16 vaccine. Following negotiations by the Africa CDC with Bavarian Nordic and EU‑HERA, the first shipment of 99,000 MVA‑BN doses arrived in DRC on 5 September with an additional 109,000 arriving on 7 September [4]. In addition, US President Joe Biden has announced the donation of an additional 1 million mpox doses and $500 million on 24 September [17].

Efforts are underway to ensure proper cold chain management, in‑country logistics, and staff training to administer the vaccines. The Africa CDC supports the Ministry of Health in developing a pharmacovigilance system to monitor vaccines for adverse events following immunization. To support this, the Africa CDC has deployed a senior technical officer for pharmacovigilance from Addis Ababa, Ethiopia, and a technical officer from Central Africa Regional Collaborating Center in Libreville, Gabon, to support the Continental Incident Management Support Team in Kinshasa. In addition to bilateral vaccine donations, the Africa CDC is collaborating with WHO, GAVI, UNICEF, and other multilateral organizations to expedite prequalification, emergency use authorization, and the rollout of the LC16 vaccines to reach at least 10 million people across the multicounty outbreak by 2025 [5].
